# Exploring a role for Chemerin in the cardiovascular and musculoskeletal benefits of intradialytic exercise in the hemodialysis population

**DOI:** 10.1371/journal.pone.0321497

**Published:** 2025-06-25

**Authors:** Mingyue Deng, Daniel S. March, Darren R. Churchwood, Hannah M. L. Young, Patrick J. Highton, Matthew J. Denniff, Matthew P. M. Graham-Brown, James O. Burton, Luke A. Baker

**Affiliations:** 1 School of Biological Science, University of Leicester, Leicester, United Kingdom; 2 International Medical College, Chongqing Medical University, Chongqing, China; 3 Department of Cardiovascular Sciences, University of Leicester, Leicester, United Kingdom; 4 Leicester Biomedical Research Centre (BRC), Leicester Diabetes Centre, University of Leicester, Leicester, United Kingdom; 5 National Institute for Health and Care Research Applied Research Collaboration (ARC) East Midlands, University of Leicester, Leicester, United Kingdom; 6 Department of Respiratory Sciences, University of Leicester, Leicester, United Kingdom; University of Pretoria Faculty of Health Sciences, SOUTH AFRICA

## Abstract

**Background:**

Cardiovascular disease is the leading cause of death for people receiving hemodialysis. Intradialytic cycling (IDC) has been shown to improve cardiovascular health in the hemodialysis population, but specific mechanisms require elucidation. Chemerin is an adipokine which contributes to the inflammatory process and may be associated with the cardiovascular benefits of IDC and physical function in hemodialysis population.

**Methods:**

Adults undertaking ≥3 months hemodialysis were randomized to either IDC (30 min each time, moderate intensity, thrice weekly) and usual care; or usual care only (control group). 88 blood samples were retrospectively analyzed for chemerin concentrations using ELISA. Unadjusted and adjusted linear regression was used to understand how changes in chemerin are associated with changes in cardiovascular and musculoskeletal health in response to IDC.

**Results:**

There was a significant increase of plasma chemerin concentration after 6 months in both groups. A positive association was detected between chemerin and short physical performance battery at baseline (β = 0.264, p = 0.017). There was no correlation of chemerin with cardiovascular, body composition, and other physical function markers.

**Conclusions:**

This study is the first to show plasma level of chemerin increases with time on hemodialysis. No evidence was found to support a role for chemerin in modifying cardiac structure and function in people undertaking IDC. Further studies should investigate the associations between chemerin and physical performance.

## Introduction

Cardiovascular disease (CVD) is the leading cause of death for people with end-stage kidney disease (ESKD) receiving hemodialysis [1–3]. Studies have concluded systemic inflammation, endothelial activation, vascular calcification, accumulation of gut microbiota-derived uremic toxins and anemia as non-traditional risk factors of CVDs in hemodialysis population [3,4]. As a result of the pro-inflammatory state within this population (through factors such as the dialysis process itself) and the additional stress to endotoxin exposure, the internal antioxidant system is impaired [3,5]. The chronic inflammatory environment is characterized by elevated levels of intracellular reactive oxygen species (ROS) and circulating inflammatory factors [5], which contribute to vascular and myocardial remodeling in this population. Recently, A 6-month intradialytic cycling (IDC) intervention has been shown to improve significant cardiovascular structure and function in the hemodialysis population [2], though the specific mechanisms that drove these adaptations are not known.

Chemerin, a novel adipokine predominantly secreted from the liver and white adipose tissue in its precursor form [6,7], is cleaved into different bio-active forms [8]. Research has shown it elicits its biological effects via 3 G-protein-coupled receptors, in which chemokine-like receptor 1 (CMKLR1/ChemR23) is suggested to be the main receptor of action [9–11]. Over-activated chemerin/ChemR23 axis has been shown to disturb the function of endothelial cells, vascular smooth muscle cells, and perivascular adipose tissue by inducing proliferation, lipid deposition and inflammation, contributing to the development of CVD risk factors in chronic kidney disease (CKD) [3,11,12]. However, a significant survival advantage brought by increased chemerin has been reported in dialysis population, which persisted after adjustment for inflammation [13]. This may be related with the vascular protective potential of chemerin/ChemR23 signaling [14], though further investigation is required to understand the relationship between chemerin and cardiovascular factors in the hemodialysis population. Previous work suggests circulatory chemerin concentrations are associated with physical inactivity in metabolic disorders, and a suppressive effect of exercise on circulating chemerin has been reported in patients with metabolic disorders including obesity and type 2 diabetes [15]. It may impair glucose uptake, induce insulin resistance, contribute to excessive levels of pro-inflammatory cytokines and mitochondrial ROS in skeletal muscle, all of which lead to exacerbation of muscle wasting [16,17]. However, we do not know the relationship between chemerin and physical function in the hemodialysis population.

Therefore, this retrospective analysis of the CYCLE-HD study aimed to investigate the association between chemerin and the CV benefits noted in response to IDC. As a secondary analysis, we investigated a role for chemerin in skeletal muscle mass, function and body composition in hemodialysis population.

## Methods

### Participants

Individuals receiving hemodialysis (>3 months) ≥18 years of age were eligible for inclusion. The CYCLE-HD trial was given ethical approval by the National Health Service (NHS) Research Ethics Committee East Midlands (Northampton, UK; Ref: 14/EM/1190) and was conducted according to the Declaration of Helsinki. All participants provided written informed consent and the trial recruitment period was from March 2015 until April 2018, demographic and clinical characteristics were collected at the point of recruitment to align with trial-specific outcome measures.

### Study design

This study was a retrospective analysis of the 6-month CYCLE-HD trial (ISRCTN1129707). Detailed descriptions of the trial design, including randomization, specific inclusion and exclusion criteria and data collection procedures, are described previously [18]. Briefly, in an open-label, blinded endpoint, cluster randomized controlled trial, adults undergoing maintenance hemodialysis were received either a 6-month structured, progressive program of IDC and usual care (IDC group) or usual care only (control group). Participants in IDC group used specially adapted and calibrated cycle ergometers (Letto series; Motmed, Reck, Germany) 3 times a week, aiming for 30 minutes of continuous cycling at a Rating of Perceived Exertion of between 12–14. Resistance was adjusted by increasing the gears on the bike as required to progress training.

### Quantification of plasma Chemerin

Blood samples were collected from the arterial dialysis needle before each dialysis (i.e., after the short interdialytic break) and centrifugated at 2500 × *g* for 15 min at 20°C. Plasma was stored at −80°C prior to analysis of chemerin. Plasma level of chemerin was quantified using the Colorimetric ELISA Kit (Human Chemerin Quantikine ELISA; R&D Systems, Minneapolis, MN, USA) according to the manufacturer’s instructions. All reagents, including diluted sample (1:100), were prepared. Wavelength of microplate reader was set to 450 nm first and then to 570 nm to get the first and second reading results for the correction of optical imperfections. The quantification was performed in February 2024 and the coefficient of variation between analysis plates was deemed to be within acceptable ranges.

### Outcome acquisition

Bioimpedance spectroscopy (BIS) (BCM Fresenius Medical Care, Bad Homburg, Germany) was used to measure body composition before dialysis. The short physical performance battery (SPPB) (which includes gait speed along with the sit-to-stand 5 and balance test) and 60-s sit-to-stand (STS60) test were performed on a non-dialysis. Physical activity was measured using the SenseWear Pro3 Armband (BodyMedia, Pittsburgh, PA, USA), an accelerometer with physiological sensors. Participants were instructed to wear the accelerometer for 7 days on the upper arm (the opposite arm of their vascular access). Steps per day were calculated as the average for total days worn (i.e., the total number of steps divided by days worn). All participants underwent cardiac magnetic resonance imaging on a 3-T platform (Skyra; Siemens Medical Imaging, Erlangen, Germany). Prognostically important measures of cardiovascular structure and function were acquired, including: LV mass index (ratio of LV mass to body surface area); LV Mass/Left Ventricular End Diastolic Volume (LVM/LVEDV); LV Ejection Fraction (LVEF), Global Native T1 (measure of myocardial fibrosis and inflammation); and Pulse Wave Velocity (PWV) with methods for acquisistion as previously described [2,18–20].

### Statistical analysis

The data were presented as mean ± standard deviation (SD) or number (percentage). The normality was checked by ‘p value’ from the Shapiro-Wilk test. The changes of plasma chemerin, measure of CVD, body composition and physical function markers following the 6-month intervention were obtained from data at 6 months minus baseline data. Difference of change value of chemerin between groups was measured by a linear regression with baseline value of chemerin and groups as independent variables. Unadjusted linear regression of chemerin with CV markers, body composition and physical function were performed at baseline and in the change of 6 months. Significant associations of chemerin were further explored using a multiple linear regression, adjusting for age, sex, body mass index (BMI), hemoglobin, dialysis vintage, ultrafiltration volume, history of diabetes and CVDs. All statistical analyses were performed using the Statistical Package for the Social Sciences program (SPSS for Windows, version 29; Chicago, IL, USA) and figures were produced on GraphPad Prism version 10 (GraphPad Software, San Diego, CA, USA). A p < 0.05 was considered statistically significant.

## Results

The CYCLE-HD trial enrolled from March 2015 to April 2018. This retrospective analysis was undertaken on n = 88 participants for whom we had remaining pre and post-intervention plasma blood samples for (IDC group, n = 46; control group, n = 42) (there were n = 130 reported in the primary CYCLE-HD report [2]). Demographic data is presented in Table 1. The control group was older (p = 0.049), and there was a higher percentage of males than the IDC group (p = 0.111) (Table 1).

**Table 1:** Baseline demographic characteristics of participants.

Variable	IDC group (n = 46)	Control group (n = 42)
Age, yr	53.9 ± 15.2	60.1 ± 13.7
Sex		
Female	17 (36.96)	9 (21.43)
Male	29 (63.04)	33 (78.57)
Ethnic		
White	24 (52.17)	18 (42.86)
Asian or Asian British	15 (32.61)	17 (40.48)
Black or Black British	2 (4.35)	5 (11.90)
Mixed	2 (4.35)	0 (0)
Other	3 (6.52)	2 (4.76)
Height, cm	168.6 ± 9.3	166.6 ± 11.6
Weight, kg		
Pre-weight	78.1 ± 16.4	79.3 ± 22.5
Post-weight	76.8 ± 16.1	77.9 ± 22.1
Target-weight	76.1 ± 15.9	77.5 ± 21.9
Pre-BMI	27.5 ± 5.8	28.4 ± 6.4
Ultrafiltration volume	2.1 ± 0.8	2.2 ± 0.8
Dialysis vintage, yr	2.0 ± 2.1	2.2 ± 2.8
Smoke		
Current smoker	8 (17.39)	6 (14.29)
Ex-smoker	17 (36.96)	15 (35.71)
Non-smoker	19 (41.30)	15 (35.71)
History of CVDs	34 (73.91)	36 (85.71)
Diabetes	13 (28.26)	20 (47.62)
Musculoskeletal disorder	0 (0)	6 (14.29)
Respiratory disease	1 (2.17)	1 (2.38)
Rheumatic disease	0 (0)	2 (4.76)

Data were given as mean ± SD or n (%). BMI, body mass index. Weight was measured on a nondialysis day. All participants received hemodialysis for 4 hours, 3 times a week. The dialysis surface area and the dialysate concentrations were individualized (and therefore heterogeneous) for participants. History of CVDs refers to one or more of peripheral vascular disease, atrial fibrillation, myocardial infarction, ischemic heart disease, heart failure, stroke/trans-ischemic attack and hypertension.

### Change in plasma chemerin following a 6-month intradialytic exercise program

**Fig 1:**
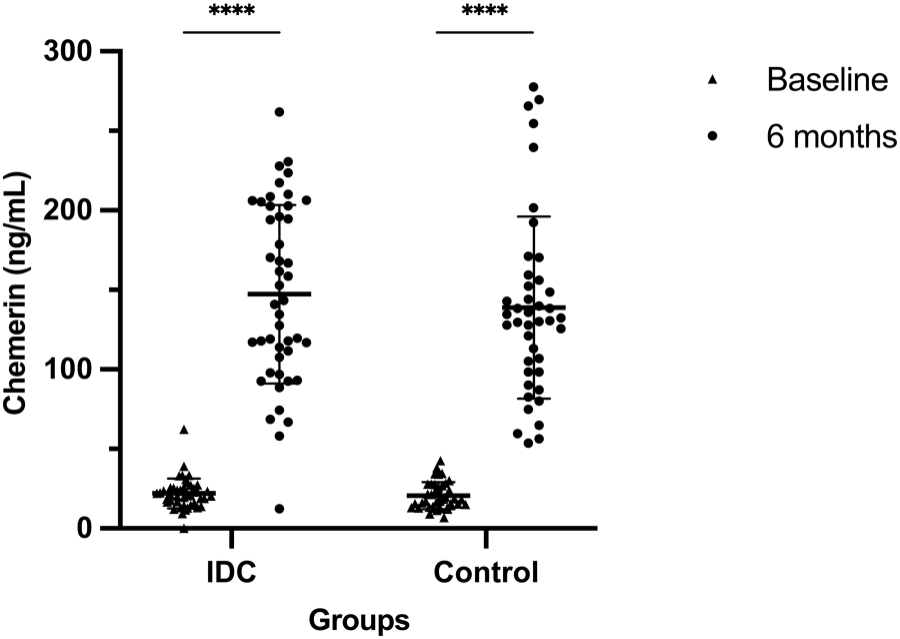
Plasma chemerin levels for IDC (n = 46) and control (n = 42) participants at baseline and 6 months. The scatter dot plots were lined at mean with SD. ****P < 0.0001.

The plasma concentration of chemerin was increased in both IDC and control groups after 6 months (p *< *0.0001) (Fig 1). There was no significant difference in the concentration change (p = 0.449) between groups in response to the 6-month program. There was no significant correlation between baseline chemerin level and its change value following 6-month program between groups (F = 0.529, p = 0.469) (Table 2).

**Table 2:** Linear regression of chemerin between change value and baseline level between groups.

Variables	F	df	p value
Randomization groups	0.269	1	0.605
Chemerin at baseline, ng/mL	0.529	1	0.469

df: degrees of freedom. Randomization groups refer to IDC and control groups. The changes following the 6-month intervention were obtained from data at 6 months minus baseline data.

### Associations of chemerin with markers of CVD, body composition and physical function

**Fig 2:**
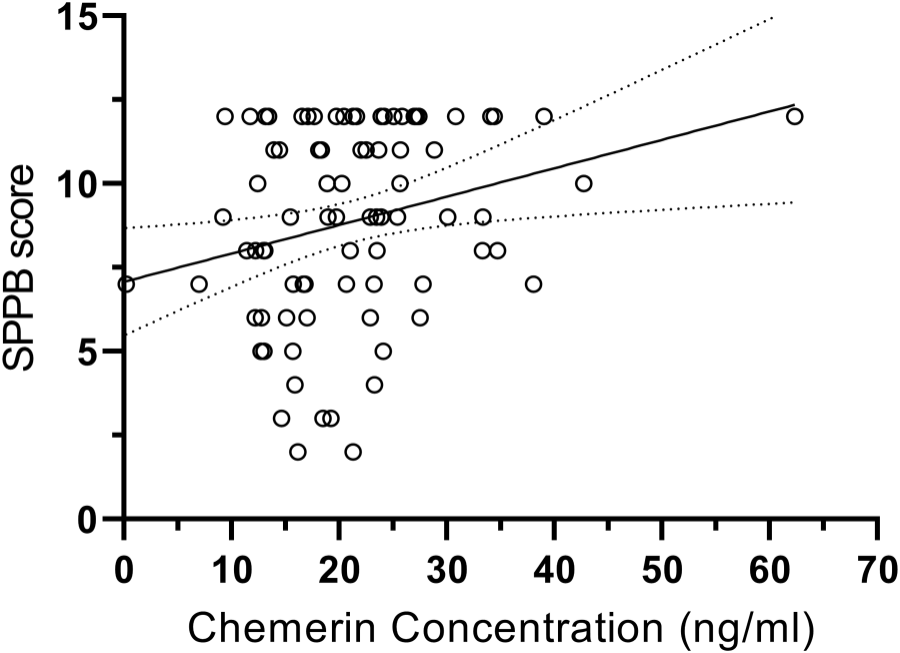
Scatter plot of the unadjusted correlation between SPPB and chemerin at baseline. There was a significant correlation between chemerin and SPPB in all participants at baseline. SPPB, short physical performance battery.

There was a significant correlation between chemerin and SPPB in all participants at baseline (β = 0.264, p = 0.017) (Table 3) (Fig 2). After adjustment for covariates including age, BMI, sex, hemoglobin, dialysis vintage, ultrafiltration volume, presence of diabetes and history of CVD, the correlation was no longer significant (β = 0.127, p = 0.245) (Table 4). There was no significant correlation of chemerin with CVD, body composition and other physical function markers at baseline and in the change value over 6 months (Table 3).

**Table 3:** Unadjusted linear regression of chemerin with CVD, body composition and physical function markers at baseline.

Variable	Standardized coefficients β (95% CI)	p value
**Baseline data of both groups**		
**Measure of cardiovascular structure and function**		
LV mass index (n = 88)	0.122 (−0.039 - 0.144)	0.260
LVM/LVEDV (n = 88)	−0.024 (−13.722 - 11.017)	0.828
LVEF (n = 88)	−0.057 (−0.238 - 0.138)	0.598
Global Native T1 (n = 86)	−0.156 (−0.083 - 0.013)	0.154
PWV (n = 78)	−0.019 (−0.461 - 0.543)	0.870
SBP (n = 85)	0.007 (−0.001 - 0.001)	0.953
DBP (n = 85)	−0.022 (0 − 0)	0.843
**Body composition**		
LTM (n = 86)	0.169 (−0.037 - 0.311)	0.122
FM (n = 86)	−0.014 (−0.143 - 0.126)	0.898
**Physical function**		
SPPB (n = 82)	0.264 (0.149 - 1.494)	0.017*
STS60 (n = 82)	0.137 (−0.066 - 0.279)	0.223
Gait Speed (n = 82)	−0.125 (−0.901 - 0.254)	0.268
Steps per day (n = 77)	0.125 (0 - 0.001)	0.280
**Change value following 6 months of IDC group**		
LV mass index (n = 46)	−0.055 (−2.844 - 1.970)	0.716
LVM/LVEDV (n = 46)	−0.163 (−176.313 - 52.382)	0.281
Global Native T1 (n = 43)	−0.059 (−0.515 - 0.352)	0.706
Steps per day (n = 38)	0.044 (−0.010 - 0.012)	0.794

Standardized coefficients β and p value from the regression analysis. CI: Confidence interval. *p denotes significant association. The changes following the 6-month intervention were obtained from data at 6 months minus baseline data. There was a weak correlation between chemerin and SPPB at baseline. Markers, including LV mass index, LVM/LVEDV, Global Native T1 and steps per day were selected as independent variables in the linear regression of change chemerin value due to their significant difference of change value between groups. No significance in the correlation of chemerin with measure of CVD, body composition and components of SPPB either for all participants at baseline or IDC group in the change value over 6 months. LV mass index, left ventricular mass index; LVM/LVEDV, left ventricular mass/left ventricular end diastolic volume; LVEF, left ventricular ejection fraction; PWV, pulse wave velocity; SBP, systolic blood pressure; DBP, diastolic blood pressure; LTM, lean tissue mass; FM, fat mass; STS60, 60-s sit-to-stand; SPPB, short physical performance battery.

**Table 4:** Association between chemerin and SPPB at baseline, adjusted for covariates.

Model summary									
R	R^2^	Adjusted R^2^	SE	R^2^ change	Durbin-Watson	ANOVA *F*	df1	df2	p value
0.564	0.319	0.232	2.526	0.319	1.595	3.689	9	71	< 0.001
**Independent variable**		**Standardized coefficients β (95% CI)**					**p value**		
Chemerin		0.127 (0 − 0)					0.245		
Age		−0.353 (−0.116 - −0.024)					0.004*		
BMI		0.066 (−0.066 - 0.128)					0.527		
Sex		−0.117 (−2.006 - 0.575)					0.273		
Hemoglobin		0.008 (−0.030 - 0.033)					0.935		
Dialysis Vintage		0.241 (0.038 - 0.700)					0.029*		
Ultrafiltration volume		−0.053 (−0.991 - 0.611)					0.637		
Diabetes		−0.285 (−3.086 - 0.295)					0.018*		
History of CVD		0.034 (−1.176 - 1.632)					0.901		

ANOVA: analysis of variance; df: degrees of freedom; SE: standard error; CI: Confidence interval. *p denotes significant association. History of CVD refers to one or more of peripheral vascular disease, atrial fibrillation, myocardial infarction, ischemic heart disease, heart failure, stroke/trans-ischemic attack and hypertension.

## Discussion

This study reported 6-month of IDC had no effect on circulating chemerin concentrations, which were seen to be elevated after 6-month on hemodialysis. There were no significant associations between circulating chemerin (both at baseline and in the change value over 6 months) and the measure of CVD or body composition. A positive association between chemerin and SPPB was shown, however, this did not remain after adjustment.

This study has found a considerable elevation in plasma levels of chemerin following 6-month of hemodialysis treatment. However, a 6-month program of IDC did not have any effect on circulating levels of chemerin. As chemerin plays a noticeable role in inflammation, this result mirrors the change of other inflammatory markers (interleukin-6, interleukin-10, tumor necrosis factor alpha and C-reactive protein) in previously published data for other circulating inflammatory markers from the CYCLE-HD trial [21] and suggests no effect of a 6-month program of IDC on the inflammatory state of those on hemodialysis [5,22]. As the molecular weight of chemerin exceeds the filtration limit of hemodialysis, circulating chemerin would be poorly cleared. This result may be the case that the length of the intervention may not be sufficient to modify circulating levels of chemerin. Furthermore, it is well understood that exercise intensity plays a key role in the activation of anti-inflammatory cytokines and as such the moderate exercise intensity (Rating of Perceived Exertion 12 ~ 14) is likely to be insufficient to induce a significant response [23]. In spite of our findings here, there is evidence that habitual physical activity may be able to modify systemic inflammation in the hemodialysis population [21].

No correlation has been detected between chemerin and prognostically significant measures of cardiac structure and function assessed with cardiac magnetic resonance imaging, which suggests that it may not play a role in the cardiovascular remodeling (Table 3). Chemerin has been implicated in the development of CVDs in CKD, such as coronary artery disease, hypertension and heart failure [3,11,12]. *in vitro* studies have showed a chemerin-induced apoptosis of murine cardiomyocytes and remodeling of vascular smooth muscle cells with the regulation of insulin and inflammatory cytokines [10,24], while chemerin has been reported as an inhibitor of vascular calcification based on the observation in CKD patients [14]. In spite of the potential paradoxical role of chemerin in the development of CVDs [11,13,25], it did not associate with any of the cardiovascular structure or function markers in this data.

To the best of our knowledge, this is the first study to identify the positive correlation between chemerin and physical performance in hemodialysis population, which is contrary to the findings of other inflammatory factors [21]. This correlation was no longer significant when adjusting for age, BMI, sex, hemoglobin, dialysis vintage, ultrafiltration volume, presence of diabetes and history of CVDs. Physical inactivity is significant associated with increased adverse CV events and mortality of hemodialysis population [26], which could also contribute to exacerbation of muscle loss, consequently leading to sarcopenia [27]. Studies have previously reported a disruption mediated by chemerin in bone metabolism and insulin signaling in muscle, which may contribute to the muscle wasting [16,17], osteoporosis [28], and associated frailty in people with metabolic disorders. Given that the systemic biological environment of hemodialysis population is complex, and is characterized by persistent inflammation, repeated disturbance in protein-energy balance, and a myriad of medications, further investigations are required to elucidate the role of chemerin in musculoskeletal complications in the hemodialysis environment [5,29]. In addition, although chemerin is mainly secreted from adipose tissue [6,7], no correlation has been detected between chemerin and body composition. Reduced glomerular filtration rate in ESKD patients would provide part of explanation for this finding [7,25] but further investigation is required to confirm or deny this hypothesis.

### Limitation and future research

Several limitations of this study should be acknowledged. Firstly, available technologies only allowed us to measure prochemerin, the circulating in-active full-length form of chemerin. Future work should look to quantify the cleaved active forms of chemerin in order to better elucidate the mechanistic effects of this adipokine and its metabolites. This would be repeated in other clinical populations where sarcopenia is prevalent to further understand the relationship between chemerin and musculoskeletal health. Future work should also look to ascertain the true origin of chemerin production in those with CKD, in order to understand both mechanism of action and potential therapeutic interventions, particularly in the context of musculoskeletal health and exercise interventions. In addition, the concentration of chemerin in local metabolic organs and its interaction with the circulating level are suggested to be taken into consideration when exploring its biological roles [6].

### Conclusion

This study has observed an increased circulatory level of chemerin over 6-month hemodialysis. No correlation has been shown to support a role of chemerin in CV benefits in response to intradialytic exercise. The significant correlation between chemerin and SPPB at baseline suggested that chemerin may be associated with physical function in the hemodialysis population. Further studies should explore the link between chemerin and musculoskeletal health in hemodialysis population in order to further understand its role in cardio/muscular health.
